# Essential role of axonal VGSC inactivation in time-dependent deceleration and unreliability of spike propagation at cerebellar Purkinje cells

**DOI:** 10.1186/1756-6606-7-1

**Published:** 2014-01-02

**Authors:** Zhilai Yang, Erwei Gu, Xianfu Lu, Jin-Hui Wang

**Affiliations:** 1 Institute of Biophysics, State Key lab for Brain and Cognitive Sciences, Chinese Academy of Sciences, Beijing 100101, China; 2 Qingdao University, Medical College, 38 Dengzhou, Shandong 266021, China; 3 Graduate School of the Chinese Academy of Sciences, Beijing 100049, China; 4 Department of Anesthesiology, The First Affiliated Hospital of Anhui Medical University, Hefei 230022, China

**Keywords:** Axon, Neuron, Action potential, Spike propagation, Purkinje cell, Cerebellum

## Abstract

**Background:**

The output of the neuronal digital spikes is fulfilled by axonal propagation and synaptic transmission to influence postsynaptic cells. Similar to synaptic transmission, spike propagation on the axon is not secure, especially in cerebellar Purkinje cells whose spiking rate is high. The characteristics, mechanisms and physiological impacts of propagation deceleration and infidelity remain elusive. The spike propagation is presumably initiated by local currents that raise membrane potential to the threshold of activating voltage-gated sodium channels (VGSC).

**Results:**

We have investigated the natures of spike propagation and the role of VGSCs in this process by recording spikes simultaneously on the somata and axonal terminals of Purkinje cells in cerebellar slices. The velocity and fidelity of spike propagation decreased during long-lasting spikes, to which the velocity change was more sensitive than fidelity change. These time-dependent deceleration and infidelity of spike propagation were improved by facilitating axonal VGSC reactivation, and worsen by intensifying VGSC inactivation.

**Conclusion:**

Our studies indicate that the functional status of axonal VGSCs is essential to influencing the velocity and fidelity of spike propagation.

## Introduction

Information flows among network neurons are fulfilled by spike propagation on the axons, signal transmission at the synapses, synaptic integration on the dendrites/soma [1-5]. The axons as a subcellular compartment play critical roles in processing neuronal codes [1], such as spike initiation [6-18], spike amplification [19,20] and spike propagation [21-24]. The patterns of axonal digital spikes constitute neuronal output codes to organize the brain functions. The amplification of axonal spikes ensures neuronal codes to be digital. The fidelity and velocity of axonal spike propagation influence the spikes to be efficient codes [25].

The secure propagation of sequential spikes toward axonal terminals has been challenged recently [23,24,26-28]. The infidelity of spike propagation occurred in the neurons that produced high frequency spikes [1,29], such as cerebellar Purkinje cells whose firing rates were up to 500 Hz [9,30-32]. Furthermore, the velocity of spike propagation presumably reduced in firing sequential spikes [1]. The infidelity of spike propagation enables some digital spikes to be lost, and the deceleration of spike propagation influences the temporal precision of neuronal digital codes. In order to secure axonal spike propagation without any loss of these digital codes and their precision, we have to figure out the mechanisms underlying spike propagation infidelity and deceleration.

The fidelity of spike propagation on the axons was influenced by membrane potential that altered VGSC kinetics [33-35], such as worsened by a depolarization and improved by a hyperpolarization [24,28]. Spike propagation was presumably triggered by local currents that raised membrane potentials to activate VGSCs [1]. Moreover, the depolarization recorded *in vivo* was classified into the steady and fluctuation patterns [36]. The steady-state depolarization inactivated VGSCs [19,33], and the hyperpolarization pulses facilitated their activation [34]. In addition, action potentials *in vivo* were sequential in nature [37-40], which affected VGSCs’ kinetics [41,42]. Therefore, the deceleration and infidelity of spike propagation may result from the alternation of VGSC functional status. We examined this hypothesis in the axons of cerebellar Purkinje cells, and found time-dependent deceleration and infidelity in spike propagation via VGSC inactivation.

## Results

The velocity and fidelity of spike propagation were measured at various time points of sequential spikes. Time-dependent changes in propagation velocity and fidelity, such as deceleration and infidelity, were hypothetically related to the functional status of voltage-gated sodium channels (VGSC). To test this hypothesis, we analyzed the changes in the velocity and fidelity of spike propagation while upregulating or downregulating VGSC’s functions. The proportional changes in VGSC dynamics vs. spike propagation denote that the deceleration and infidelity of spike propagation are controlled by VGSC’s functions.

### The time-dependent deceleration and infidelity of spike propagation on the axons of Purkinje cells

**Figure 1:**
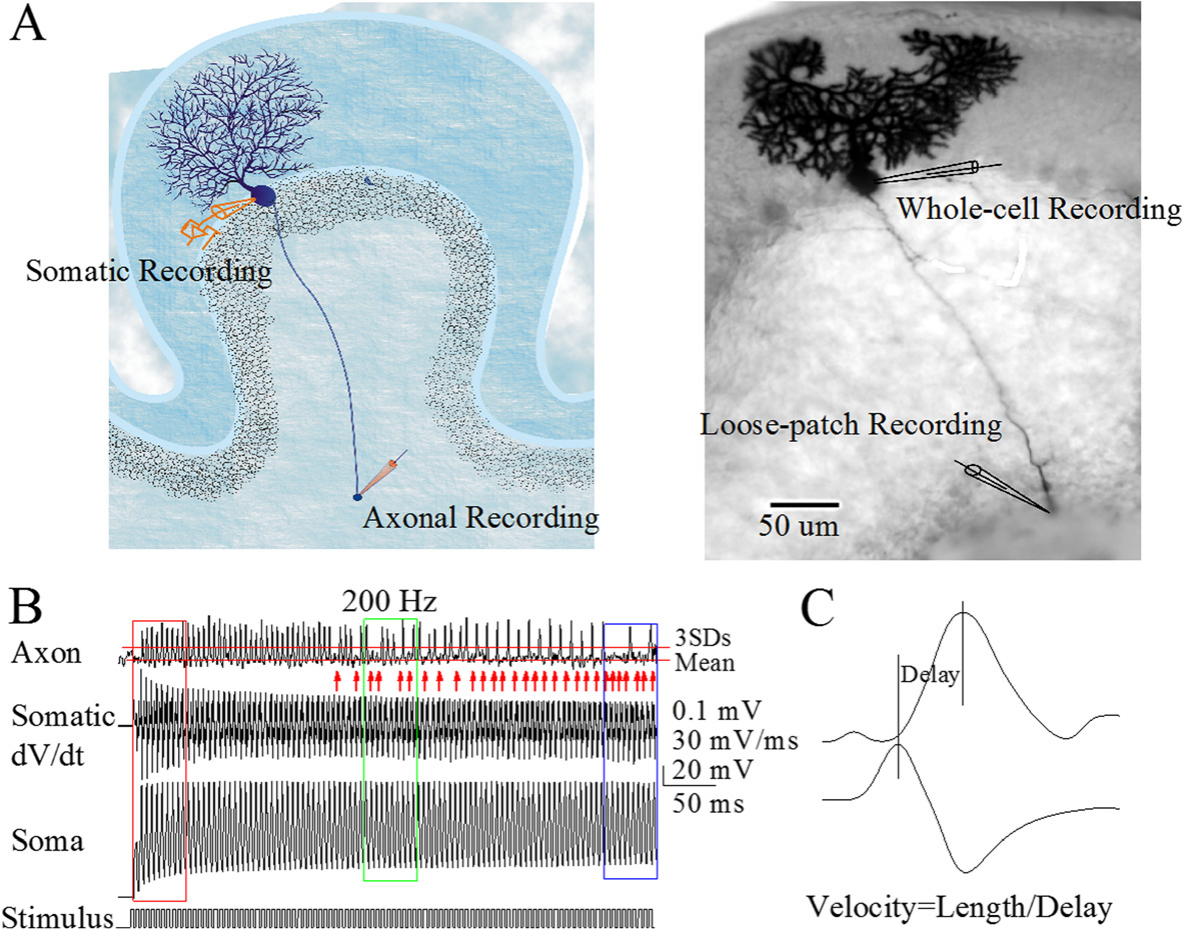
**The fidelity and velocity in the propagation of sequential spikes are measured on the main axons of cerebellar Purkinje cells (PC). A)** Left panel shows the diagram of a whole-cell recording on PC soma and a loose-patch recording on its axonal bleb. Right panel is a neurobiotin-labeled PC whose main axon extends to deep cerebellar nucleus. **B)** Top trace shows axonal spikes recorded by a loose-patch on axonal bleb, bottom trace shows spikes recorded by a whole-cell recording pipette at PC soma, and middle trace shows the dV/dt values of somatic spikes. Somatic spikes are induced by sequential depolarization pulses at 200 Hz. Spike propagation is defined as a failure if axonal spikes are lower than three times of standard deviation of mean baseline value (red lines & arrows). **C)** Top trace shows the expanded axonal spikes recorded by a loose-patch on axonal bleb and bottom trace shows the dV/dt values of somatic spikes. The difference of their peak time is called as the delay, which is used to calculate the velocity that is equal to the division of axonal length by time delay.

Spike propagation on the main axons of cerebellar Purkinje cells was measured while sequential spikes were evoked on their somata by whole-cell pipette and the propagated spikes were recorded at their axonal terminal blebs by loose-patch pipette (Figure 1A). Spike propagation fidelity was assessed by the number of spikes propagated into terminals versus the number of spikes evoked at soma [23,24,29]. Spike propagation velocity was calculated by a formula that the lengths between soma and axonal bleb were divided by peak-time from somatic spikes to axonal ones (Figure 1C). Sequential depolarization pulses were given for 0.5 second, which was based on a fact that the responses of Purkinje cells to *in vivo* stimuli lasted for seconds [43,44]. The spikes reaching to axonal terminals were accounted if their amplitudes were above the mean values plus three-times’ standard deviation of signal noise (red lines in Figure 1B; [23]).

**Figure 2:**
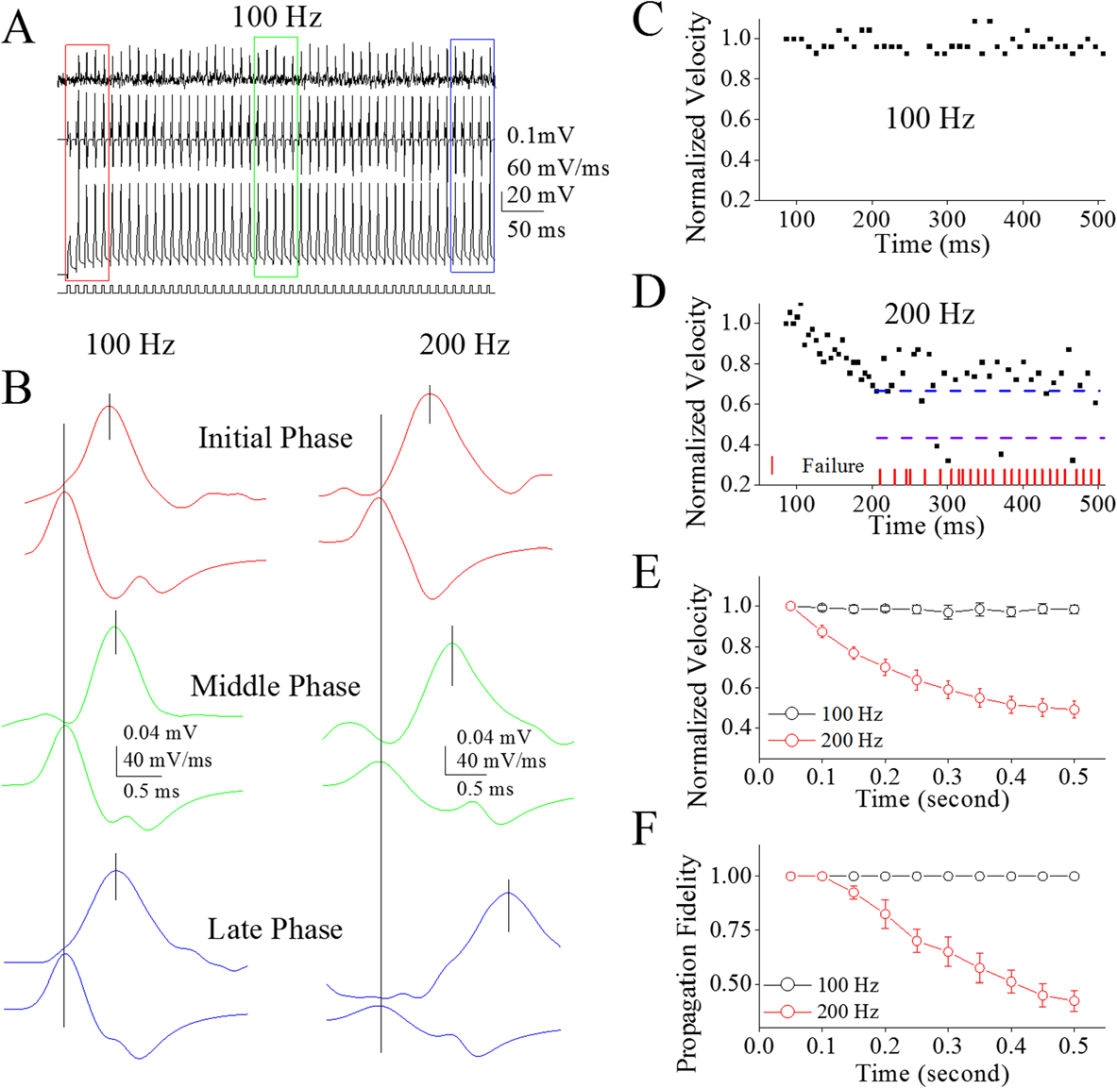
**The velocity and fidelity of spike propagation on the main axons of cerebellar Purkinje cells (PC) decrease when spiking time increases. A)** Top trace shows spikes recorded by loose-patch on axonal bleb, bottom trace is somatic spikes induced by a whole-cell recording pipette at depolarization pulses 100 Hz, and middle trace shows dV/dt values of somatic spikes. Calibration bars are 0.1 mV for axonal spikes, 20 mV for whole-cell spikes, 60 mV/ms for dV/dt and 50 ms. **B)** shows the spikes on axons (top traces) and the dV/dt of somatic spikes (bottom traces) in initial (red), middle (green) and late phases (blue) under the conditions of spike frequencies at 100 Hz (left column) and 200 Hz (right). The traces in different colors are taken from the boxes in 2**A** and 1**B** with corresponding colors. **C)** shows the normalized velocity of spike propagation vs. time at 100 Hz of spikes. **D)** illustrates the normalized velocity of spike propagation versus time at 200 Hz of spikes. The velocity of spike propagation decreases to two levels, level one (blue dash line) and level two (purple). Red vertical bars show time points of spike propagation failure. **E)** shows normalized spike propagation velocity vs. time under the conditions of spike frequencies at 100 Hz (black symbols) and 200 Hz (reds; n = 12). **F)** illustrates spike propagation fidelity (a ratio of axonal spikes to somatic ones) vs. time under the conditions of spike frequencies at 100 Hz (black symbols) and 200 Hz (reds; n = 12).

Figure 2 illustrates the effect of spiking time on spike propagation at different frequencies. The waveforms from top to bottom in Figure 2A are axonal spikes, somatic spikes’ dV/dt and somatic spikes (100 Hz), respectively. Pair waveforms in Figure 2B are the expanded axonal spikes (top) and somatic spikes’ dV/dt (bottom) in different phases (corresponding colors in Figures 2A and 1B), in which the spikes are induced by pulses at 100 (left column) and 200 Hz (right). Peak-time intervals between somatic spikes and axonal ones indicate that spike propagation delays while spiking duration prolongs and spiking frequency rises. Moreover, spike propagation is increasingly failed (red vertical bars) when spiking time prolongs (Figure 2C ~ D). In spike frequency at 200 Hz, the decrease of spike propagation velocity is ahead of the failure of spike propagation. Statistical analyses in Figure 2E illustrate the normalized velocity of spike propagation versus time at 200 (red symbols; n = 12) and 100 Hz spikes (blacks). Figure 2F shows the fidelity of spike propagation vs. time at 200 (reds; n = 12) and 100 Hz (blacks). These results indicate time- and frequency-dependent attenuation in spike propagation velocity and fidelity, i.e., spike propagation deceleration and infidelity. Parallel changes in spike propagation velocity and fidelity indicate that they may share similar mechanism.

**Figure 3:**
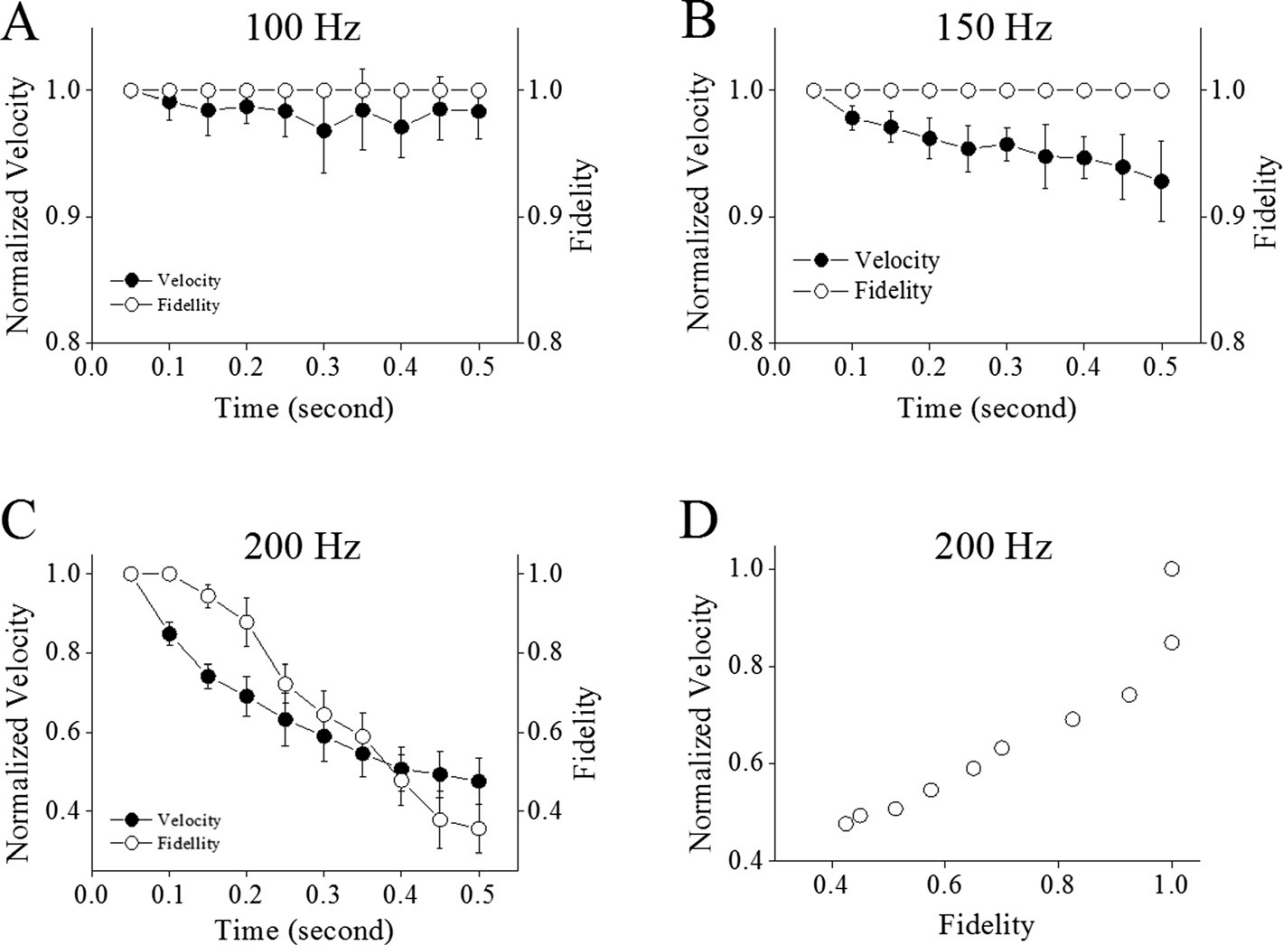
**The time-dependent deceleration and infidelity of axonal spike propagation in different spike frequencies. A)** shows the normalized velocity (filled symbols) and fidelity (opens) of spike propagation versus time when somatic spikes are induced at 100 Hz. **B)** shows the normalized velocity (filled symbols) and fidelity (opens) of spike propagation vs. time when somatic spikes are induced at 150 Hz. **C)** shows the normalized velocity (filled symbols) and fidelity (opens) of spike propagation vs. time when somatic spikes are induced at 200 Hz (n = 12). **D)** illustrates spike propagation velocity versus fidelity at 200 Hz of spike frequency. The data indicate that the deceleration of spike propagation is ahead of the infidelity.

We then analyzed whether spike propagation velocity or fidelity is more sensitive to spiking time. Figure 3 illustrates the velocity and fidelity of spike propagation versus time, in which somatic spikes are induced at different frequencies (100 in A, 150 in B and 200 Hz in C). With increases in spiking time and frequency, the attenuation of propagation velocity appears ahead of that of fidelity. Figure 3D shows the relationship between spike propagation velocity and fidelity at 200 Hz in frequency, in which their values are taken from corresponding time points in Figure 3C. These results indicate that the spike propagation velocity is more sensitive to spiking time than propagation fidelity.

**Figure 4:**
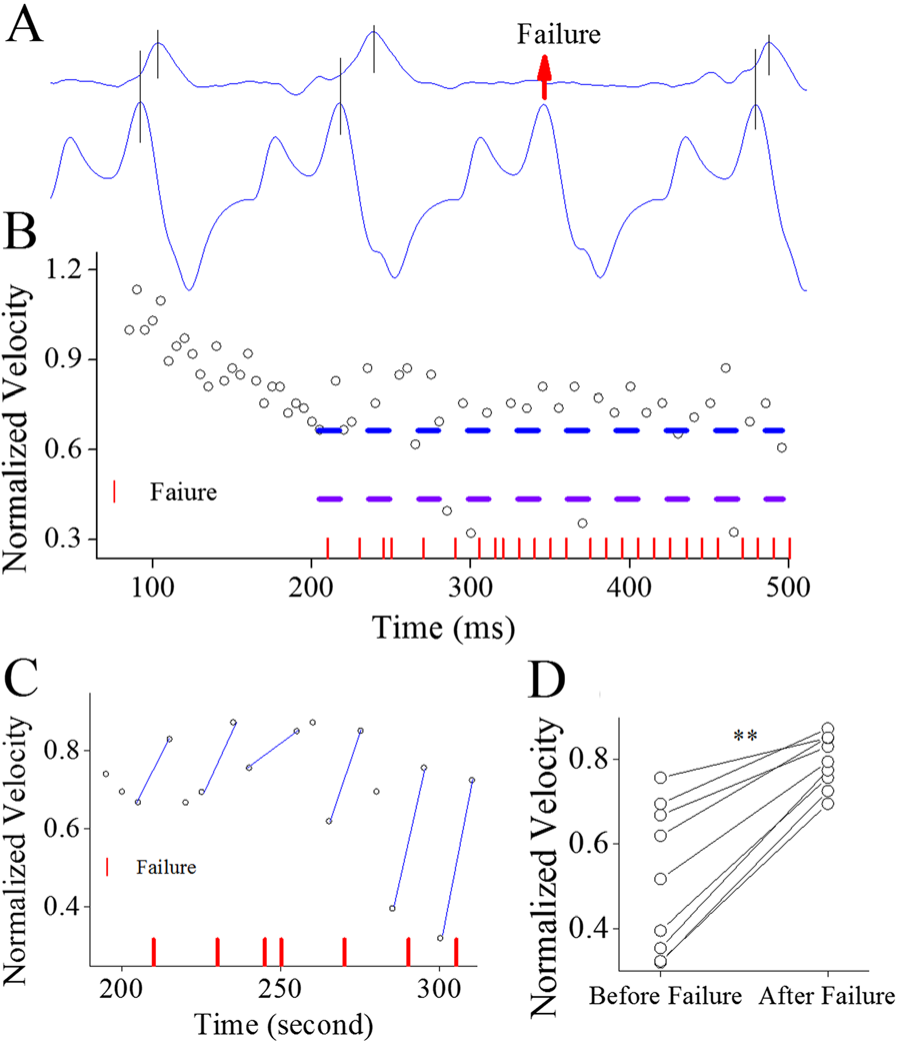
**Propagation failure of a spike makes the propagation deceleration of subsequent spike partially recovered. A)** Top trace shows axonal spikes, and bottom trace shows dV/dt values of somatic spikes. A comparison of peak time indicates a sequence that spike propagation slows down, spike propagation fails and spike propagation velocity recovers. **B)** shows normalized velocity of spike propagation versus time at 200 Hz of sequential spikes. The velocity of spike propagation decreases to two levels, i.e., initial level one (blue dash line) and subsequent level two (purple). Red vertical bars show the time points of spike propagation failure. **C)** shows normalized velocity of spike propagation vs. time, which was taken from **B)** in corresponding time. A decrease in spike propagation velocity is followed by loss of spikes during their propagation and a subsequent propagation velocity recovery (blue lines). **D)** illustrates the normalized velocity of spike propagation before and after spike propagation failure, i.e., the recovery of spike propagation deceleration. Two asterisks indicate p < 0.01.

In terms of a relationship between spike propagation velocity and fidelity, Figure 4 illustrates the changes in the velocity and fidelity of spike propagation during long-term spikes. A deceleration of spike propagation is ahead of the infidelity of spike propagation (Figure 4A ~ B). Interestingly, the attenuation of spike propagation velocity partially recovers after a spike fails to propagate (Figure 4C ~ D). Moreover, the propagation velocity appears attenuated to two levels (Figure 4B), in which level one (blue dash-line) is defined when spike failure occurs randomly and level two (purple) is defined when the propagation of subsequent spike fails absolutely. Spike propagation velocity recovers to level one from level two after it fails. The immediate recovery of propagation velocity after the propagation failure of a spike implies that the deceleration and infidelity of spike propagation may share similar mechanisms.

Spike propagation was presumably based on local currents that depolarized membrane potentials to activate VGSCs [1,21-24,33,35]. We focused on studying the roles of VGSC’s functional status in the deceleration and infidelity of spike propagation on the axons of cerebellar Purkinje cells.

### Facilitating VGSC’s reactivation reverses the time-dependent deceleration and infidelity of spike propagation

The facilitation of VGSC reactivation was done by two approaches, i.e., the prevention of VGSC inactivation by using anemone toxin (ATX), a blocker of VGSC inactivation [45,46], and the promotion of VGSC recovery from inactivation by hyperpolarization [34]. If VGSC functional status controls the time-dependent deceleration and infidelity of spike propagation, the facilitation of VGSC reactivation should block these phenomena. Moreover, the values of dV/dt for spike rising phase (the change of spike potentials per time unit) were thought to be the indices of VGSC’s activation and reactivation [47,48]. We also measured the changes of spike’s maximal dV/dt during long-term spiking.

**Figure 5:**
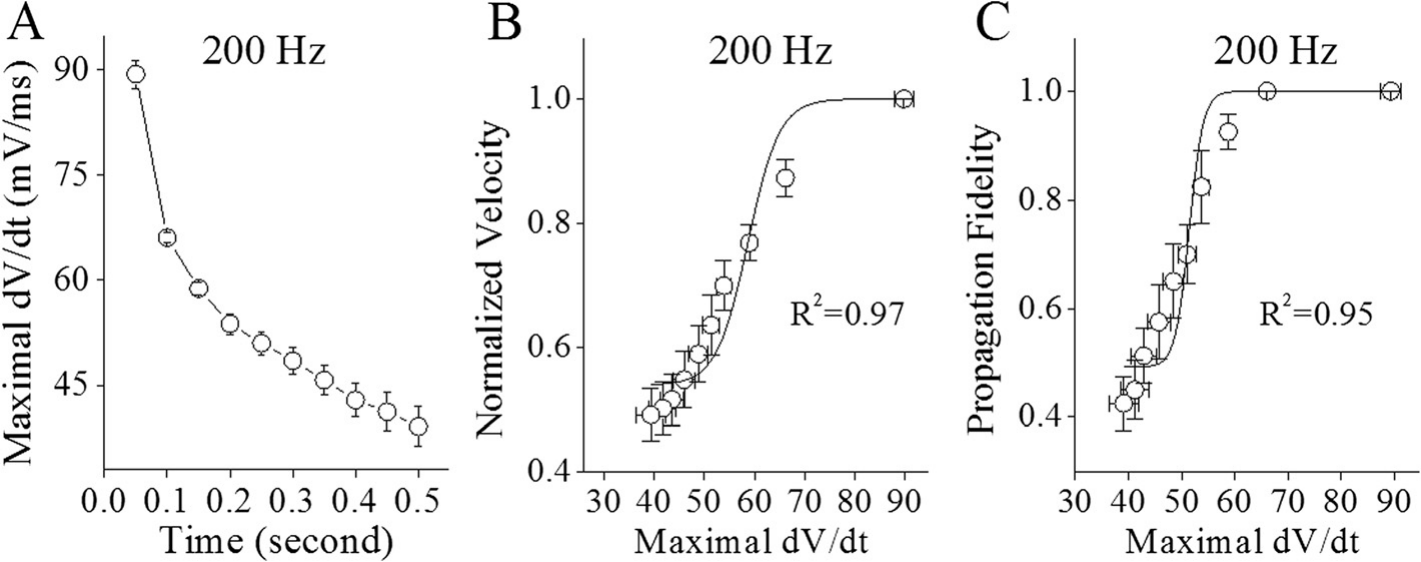
**The proportional correlations between maximal dV/dt vs. spike propagation fidelity or velocity indicate an involvement of VGSCs in spike propagation. A)** illustrates maximal dV/dt vs. spiking time at cerebellar Purkinje cells when somatic spikes are induced at 200 Hz. **B)** shows a proportional relationship between normalized spike propagation velocity and maximal dV/dt, where the data of spike propagation velocity are from red symbols in Figure 2E. **C)** shows proportional relationship between spike propagation fidelity and maximal dV/dt, in which the data of spike propagation fidelity are from red symbols in Figure 2F.

Figure 5 illustrates the dynamical changes of maximal dV/dt values when the axons of cerebellar Purkinje cells propagate sequential spikes. The maximal dV/dt values appear reduced during long-lasting spiking (Figure 5A; n = 12). By plotting spike propagation velocity or fidelity versus maximal dV/dt in corresponding time points, we observed the proportional correlations between the normalized velocity of spike propagation and maximal dV/dt (Figure 5B, Boltzmann’s fitting) or between the fidelity of spike propagation and maximal dV/dt (Figure 5C). These results indicate that the ability of VGSC reactivation influences the velocity and fidelity of spike propagation.

**Figure 6:**
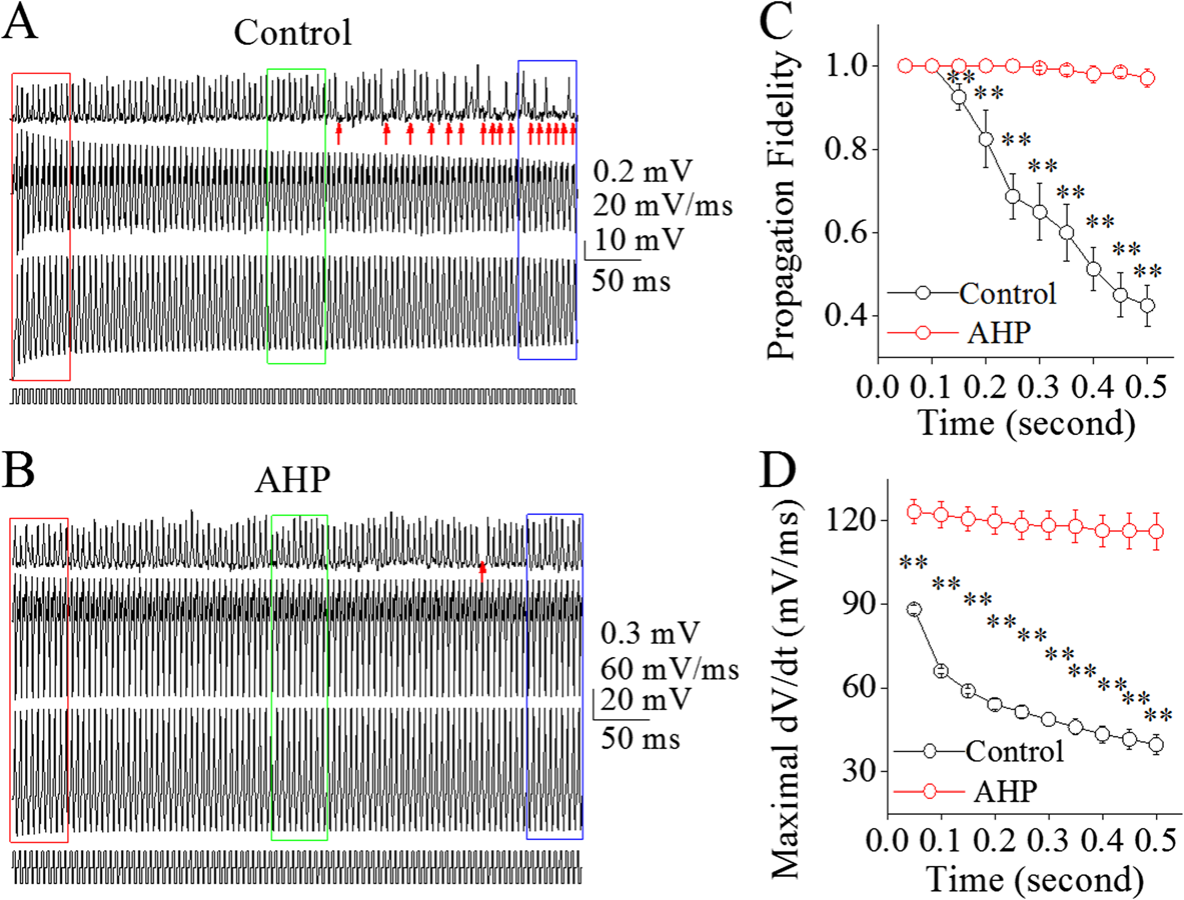
**Afterhyperpolarization (AHP) improves the time-dependent infidelity of spike propagation on the main axons of cerebellar Purkinje cells. A)** Top trace illustrates spikes recorded by a loose-patch on axonal bleb, bottom trace is somatic spikes induced by a whole-cell recording pipette at depolarization pulses (200 Hz) and middle trace shows dV/dt values of somatic spikes under control. The arrows under axon-recorded signals show the failure of spike propagation. Calibration bars are 0.2 mV for axon spikes, 10 mV for whole-cell spikes, 20 mV/ms for dV/dt and 50 ms. **B)** Top trace illustrates spikes recorded on axonal bleb, bottom trace is somatic spikes induced by depolarization pulses (200 Hz) and middle trace shows dV/dt values of somatic spikes under the condition of giving AHP (biphasic pulses, depolarization plus hyperpolarization in bottom trace). An arrow under axon-recorded signals shows a spike propagation failure on axon. Calibration bars are 0.3 mV for axonal spikes, 20 mV for whole-cell spikes, 60 mV/ms for dV/dt and 50 ms. **C)** shows spike propagation fidelity vs. somatic spiking time under the conditions of control (black symbols) and biphasic pulses (reds; two asterisks, p < 0.01; n = 9). **D)** shows maximal dV/dt vs. spiking time under the conditions of control (black symbols) and AHP (reds; asterisks, p < 0.01; n = 9).

**Figure 7:**
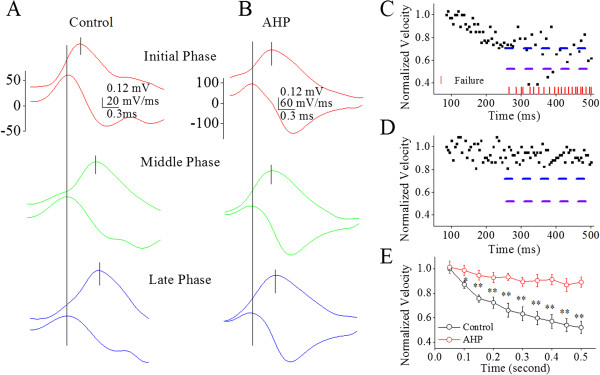
**Afterhyperpolarization (AHP) improves a time-dependent deceleration of spike propagation on the main axons of cerebellar Purkinje cells. A ~ B)** shows the spikes on axons (top traces) and dV/dt of somatic spikes (bottom traces) in initial (red), middle (green) and late phases (blue) under conditions of control **(A)** and biphasic pulses **(B)**. The traces in different colors are taken from the boxes in 6**A** ~ **B** with corresponding colors. **C)** illustrates the normalized velocity of spike propagation versus time at 200 Hz of spikes under controls. The velocity of spike propagation decreases to two levels, level one (blue dash line) and level two (purple). Red vertical bars illustrate the time points of spike propagation failure. **D)** shows the normalized velocity of spike propagation vs. time at 200 Hz of spikes when biphasic pulses (AHP) is given. **E)** shows the normalized velocity of spike propagation vs. somatic spiking time under the conditions of control (black symbols) and biphasic pulses (reds; asterisks, p < 0.01; n = 9).

We subsequently examined the roles of hyperpolarization in regulating spike propagation velocity and fidelity since a hyperpolarization promoted VGSC recovery from inactivation [34]. Figure 6 shows the effect of hyperpolarization on spike propagation fidelity. Hyperpolarization appears to attenuate the failure of spike propagation (Figure 6A ~ B). The failure of propagating sequential spikes at 200 Hz (black symbols in Figure 6C) is significantly prevented by hyperpolarization (red symbols; n = 9, p < 0.01). In the meantime, the decrease of maximal dV/dt values during long-term spiking (black symbols in Figure 6D) is reversed by hyperpolarization (red symbols; n = 9, p < 0.01). Moreover, Figure 7 shows the effect of hyperpolarization on spike propagation velocity. Figure 7A ~ B shows that the hyperpolarization pulses appear to attenuate the deceleration of spike propagation. The deceleration of propagating spikes (200 Hz) at level one and level two (black symbols in Figure 7C ~ D) in this example is prevented by hyperpolarization (reds). Statistic analysis in Figure 7E shows that the deceleration of spike propagation (black symbols) is reversed by hyperpolarization (red symbols; n = 9, p < 0.01). Therefore, the upregulation of axonal VGSC’s functions by hyperpolarization secures the velocity and fidelity of propagating spikes.

**Figure 8:**
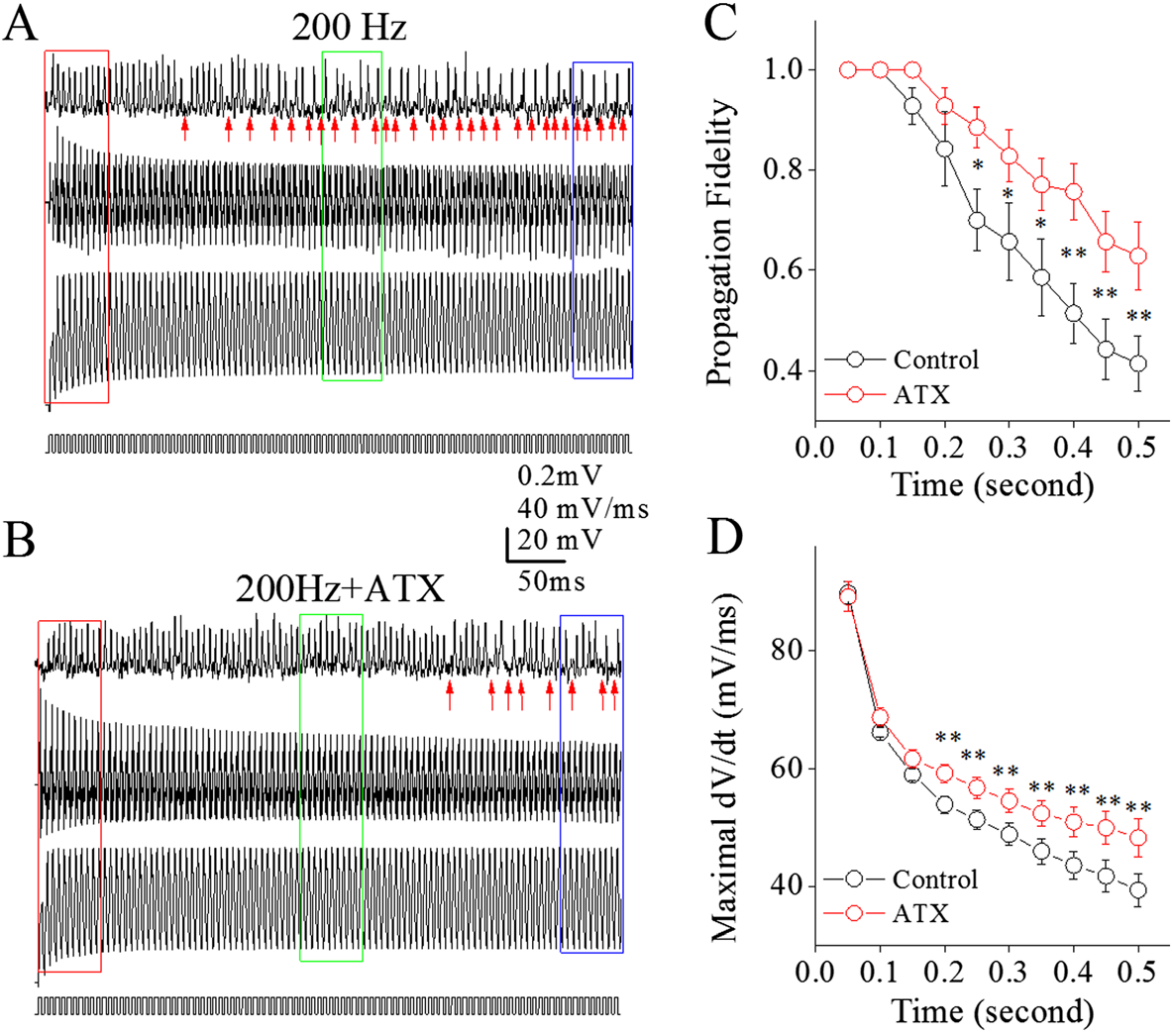
**Anemone toxin improves the time-dependent infidelity of spike propagation on the main axons of cerebellar Purkinje cells.** 5 μM ATX (a blocker of VGSC inactivation) was given to the middle point of axons between PC somata and axonal terminals. **A)** Top trace shows spikes recorded on axonal bleb, bottom trace shows somatic spikes induced by depolarization pulses (200 Hz) and middle trace shows the dV/dt values of somatic spikes under the control. Arrows under axon-recorded signals indicate the failure of spike propagation on axons. Calibration bars are 0.2 mV for axon spikes, 20 mV for whole-cell spikes, 20 mV/ms for dV/dt and 50 ms. **B)** Top trace shows spikes recorded on axonal bleb, bottom trace is somatic spikes induced by depolarization pulses (200 Hz) and middle trace shows dV/dt values of somatic spikes under the condition of applying ATX. Arrows under the axon-recorded signals shows the spike propagation failure on axon. **C)** shows spike propagation fidelity versus spiking time under the conditions of control (black symbols) and ATX (red; an asterisk, p < 0.05; two asterisks, p < 0.01; n = 8). **D)** illustrates maximal dV/dt versus spiking time under the conditions of control (black symbols) and ATX (reds; two asterisks, p < 0.01; n = 8).

**Figure 9:**
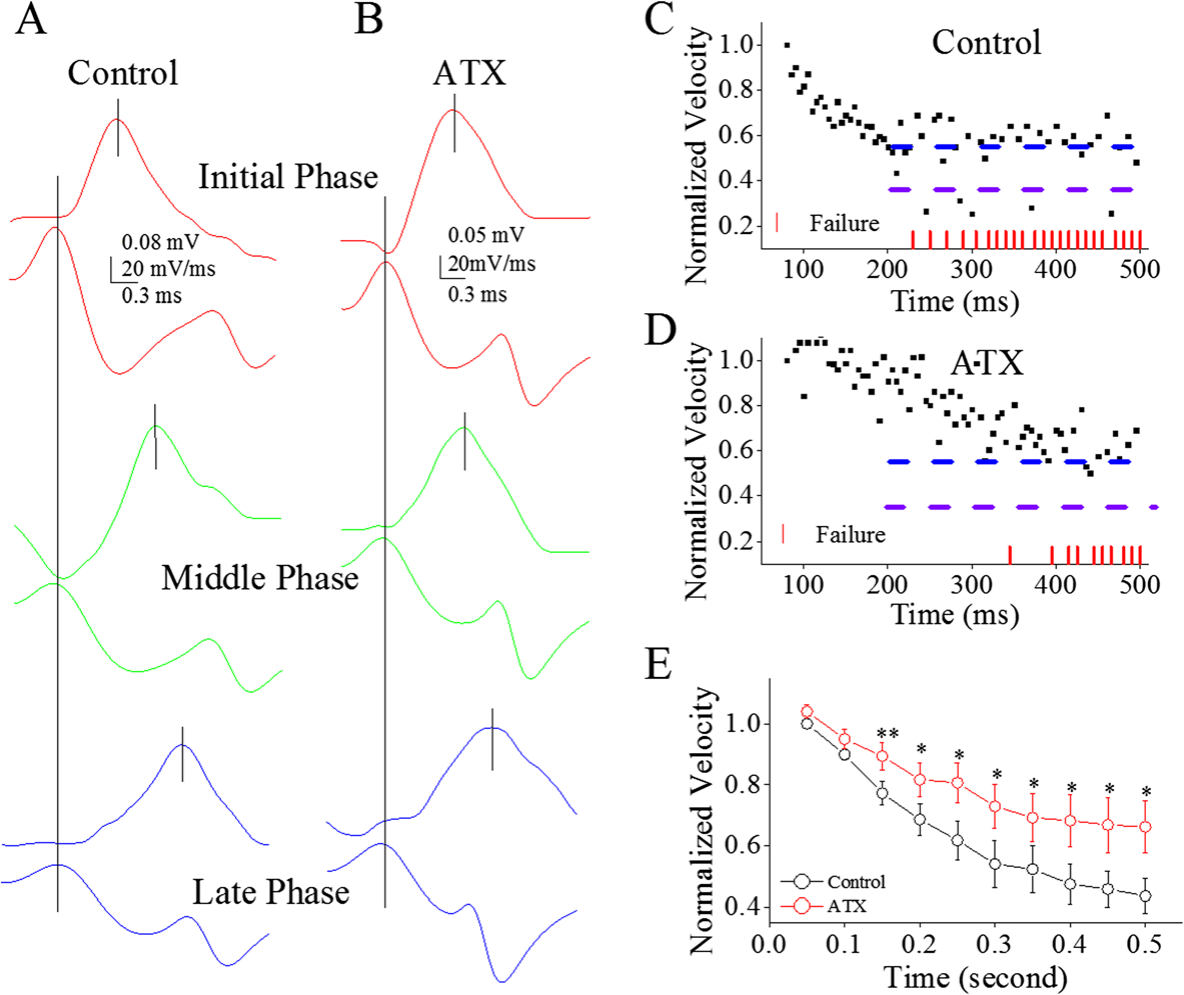
**ATX partially reverses a time-dependent deceleration of spike propagation on the main axons of cerebellar Purkinje cells. A ~ B)** shows spikes on axons (top traces) and dV/dt of somatic spikes (bottom traces) in initial (red), middle (green) and late phases (blue) under the conditions of control **(A)** and ATX **(B)**. Traces in different colors are taken from boxes in 8**A** ~ **B** with corresponding colors. **C)** illustrates the normalized velocity of spike propagation vs. time at 200 Hz of sequential spikes under the control. Spike propagation velocity decreases to two levels, level one (blue dash line) and two (purple). Red vertical bars show time point of spike propagation failure. **D)** shows the normalized velocity of spike propagation versus time at 200 Hz of spikes when ATX is given. The deceleration of spike propagation is partially reversed from level two to level one. **E)** illustrates the normalized velocity of spike propagation versus somatic spiking time under the conditions of control (black symbols) and ATX (reds; an asterisk, p < 0.05, n = 8).

Furthermore, we examined the roles of ATX in regulating spike propagation velocity and fidelity. 5 μM ATX was puffed to axonal middle points between somata and axonal blebs of Purkinje cells. Figure 8 illustrates the effect of ATX on spike propagation fidelity. ATX appears to reduce propagation infidelity (Figure 8A ~ B). The shortfall of spike propagation at 200 Hz (black symbols in Figure 8C) is partially blocked by ATX (reds; n = 8, p < 0.01). The decrease of maximal dV/dt during spike propagation (black symbols in Figure 8D) is also reversed by ATX (red symbols; n = 8, p < 0.05). In addition, Figure 9 shows the influence of ATX on spike propagation velocity. Figure 9A ~ B shows that ATX appears to reduce the deceleration of spike propagation. The deceleration of propagating spikes (200 Hz) to level two (black symbols in Figure 9C ~ D) in this example is reversed by ATX (red symbols). This reversion is statistically significant (p < 0.05, n = 8; Figure 9E). Thus, ATX secures spike propagation velocity. The upregulation of axonal VGSC dynamics by ATX secures the velocity and fidelity of propagating sequential spikes.

The results above indicate that the lowered functional status of axonal VGSCs is reason for the deceleration and infidelity of propagating sequential spikes. To be sure that the functional status of axonal VGSCs is essential to the fidelity and velocity of spike propagation, we examined whether the attenuation of VGSC function makes spike propagation deceleration and infidelity to be worsen.

### Suppressing VGSC’s reactivation worsens the time-dependent deceleration and infidelity of spike propagation

As VGSC’s inactivation is depolarization- and time-dependent [33,35], VGSC’s functional status was suppressed by depolarizing membrane potentials. Instead of applying a train of depolarization pulses to induce spikes, we applied a long-term steady depolarization to evoke sequential spikes and measured the fidelity and velocity of spike propagation.

**Figure 10:**
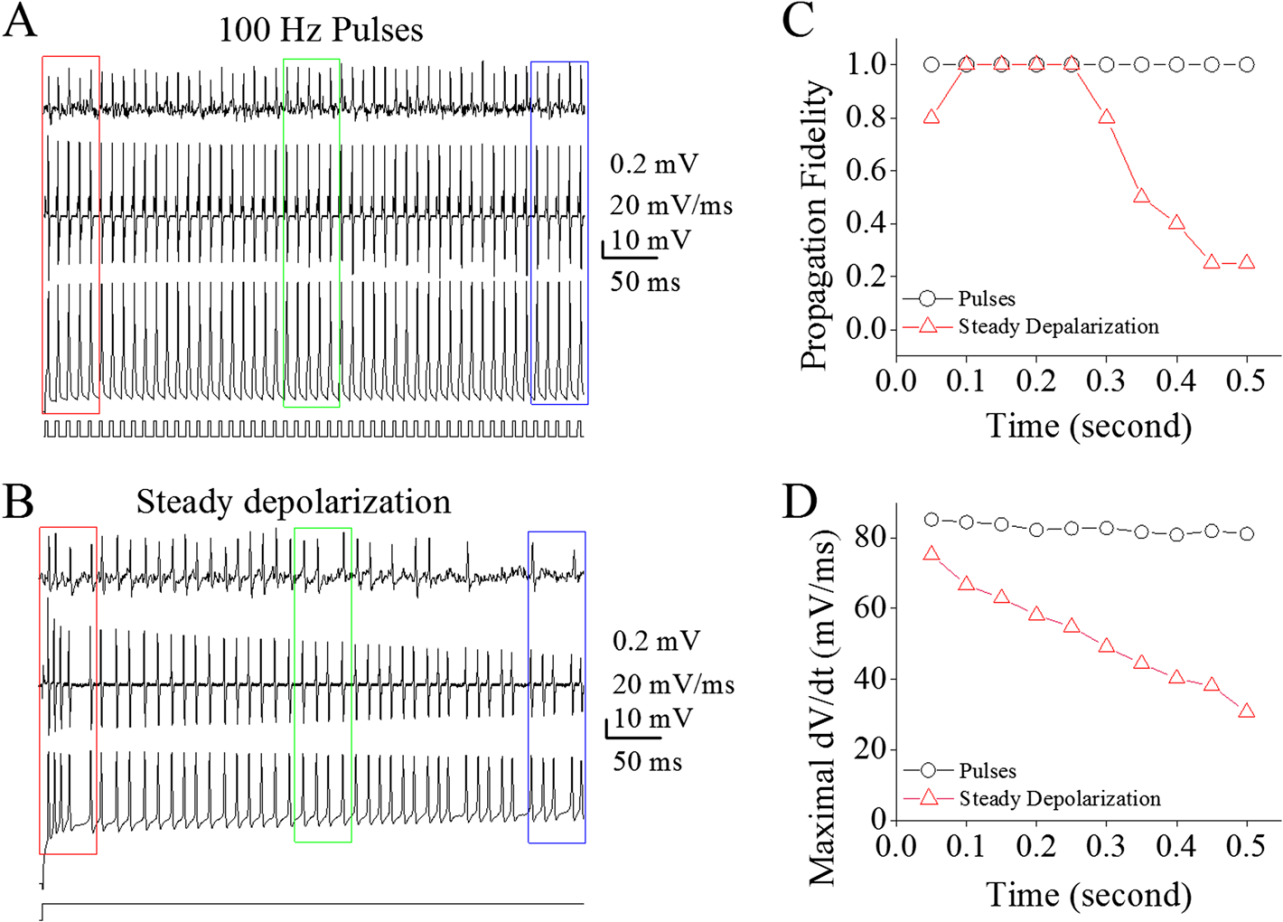
**Prolonged steady depolarization worsens the time-dependent infidelity of spike propagation on the main axons of cerebellar Purkinje cells. A)** shows spikes recorded on axonal bleb (top trace), somatic spikes induced by a train of depolarization pulse at 100 Hz (bottom) and dV/dt values of somatic spikes (middle). **B)** illustrates spikes recorded on axonal blebs (top trace), somatic spikes induced by a prolonged steady depolarization pulse (bottom) and dV/dt values of somatic spikes, in which spike’s frequency reaches to 100 Hz. Red arrows indicate the failure of spike propagation. Calibration bars are 0.2 mV for axonal spikes, 10 mV for whole-cell spikes, 20 mV/ms for dV/dt and 50 ms. **C)** illustrates propagation fidelity (a ratio of axonal spikes to somatic ones) versus spiking time by giving steady depolarization (red symbols) and depolarization pulses (black symbols). **D)** illustrates maximal dV/dt versus spiking time by giving steady depolarization (red symbols) and depolarization pulses (black symbols).

Figure 10 shows the effect of long-term steady depolarization on the fidelity of spike propagation on the axons of cerebellar Purkinje cells, in which the depolarization (0.5 second) is set on an intensity to induce spikes at 100 Hz. Comparing the spikes induced by the steady depolarization to the spikes induced by a train of depolarization pulses at 100 Hz (Figure 10A), we can see that some spikes induced by the steady depolarization (Figure 10B) fail to be propagated to the axonal terminal. Spike propagation fidelity shifts toward downside in this steady depolarization (red symbols in Figure 10C), compared to that in a train of depolarization pulses (black symbols). In the meantime, maximal dV/dt for the spikes induced by steady depolarization decrease with time (red symbols in 10D). Thus, the steady depolarization worsens the time-dependent infidelity of spike propagation by inactivating VGSCs on the axons of Purkinje cells.

**Figure 11:**
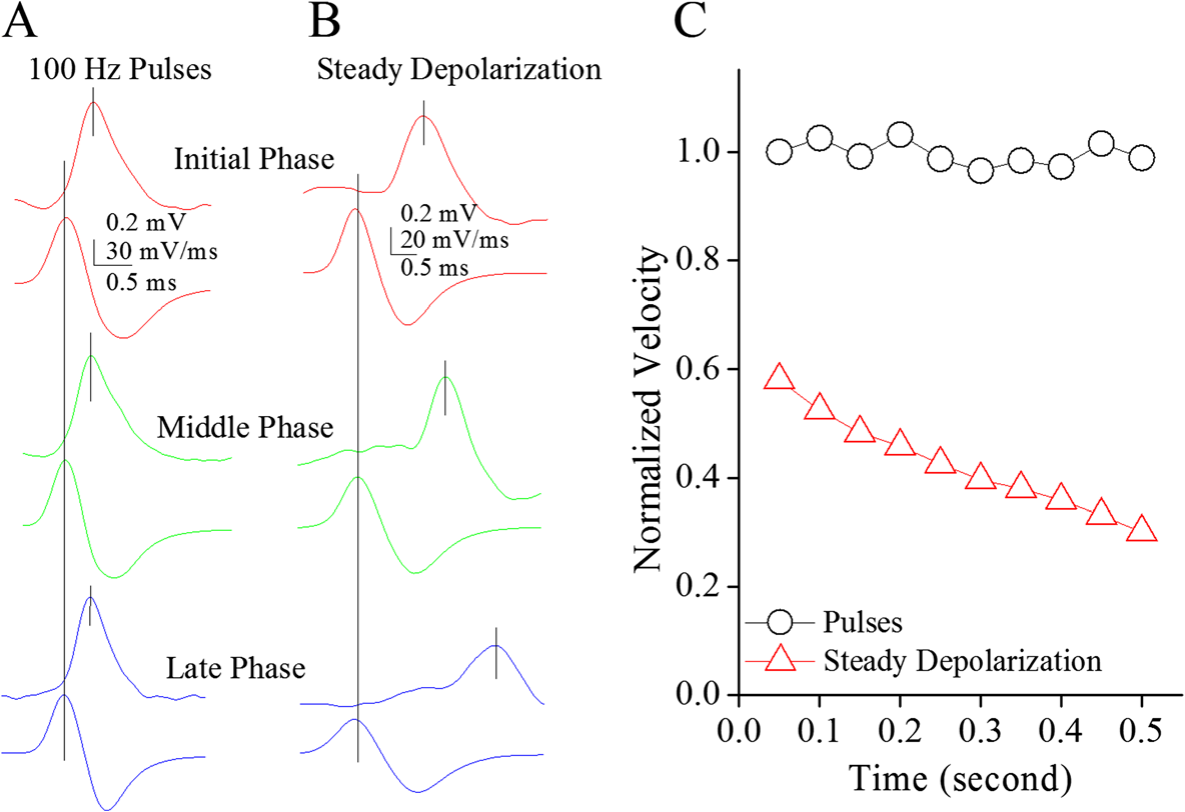
**Prolonged steady depolarization worsens the time-dependent deceleration of spike propagation on the main axons of cerebellar Purkinje cells. A)** illustrates the spikes on axons (top traces) and dV/dt of somatic spikes (bottom traces) in initial (red), middle (green) and late phases (blue) induced by a train of depolarization pulses at 100 Hz. **B)** shows the spikes on axons (top traces) and dV/dt of somatic spikes (bottom traces) in initial (red), middle (green) and late phases (blue) induced by a prolonged steady depolarization. Traces in different colors are taken from the boxes in 10**A** ~ **B** with corresponding colors. Calibration bar is 0.5 ms. **C)** illustrates the normalized velocity of spike propagation vs. spiking time by giving a steady depolarization (red symbols) and a train of depolarization pulses (black symbols).

Figure 11 shows the effect of long-time steady depolarization on the velocity of spike propagation on the axons of cerebellar Purkinje cells. The peak-time differences between axonal spikes and somatic spike dV/dt appear shorter during pulse depolarization (Figure 11A) than steady depolarization (Figure 11B). Figure 11C shows a significant lower propagation velocity of somatic spikes induced by the steady depolarization (red symbols) than by a train of depolarization pulses (black symbols). Therefore, a steady depolarization worsens the time-dependent deceleration of spike propagation by inactivating VGSCs on the axons of Purkinje cells.

## Discussion

Our study demonstrates the time-dependent infidelity and deceleration of spike propagation in the axons of cerebellar Purkinje cells (Figures 1 and 2), in which spike propagation velocity is more sensitive to spiking time (Figures 3 and 4). Propagation fidelity and velocity are proportionally correlated to spike rising slopes (Figure 5). The time-dependent infidelity and deceleration of spike propagation are improved by facilitating VGSC reactivation (Figures 6, 7, 8 and 9) and are exacerbated by inactivating VGSCs (Figures 10 and 11). Thus, the functional status of VGSCs is essential to control the propagation of digital spikes on the axons, sharing similar mechanism to their generation. These data reveals a notion that the fidelity and velocity of spike propagation depend on the time of firing sequential spikes. The influences of membrane potentials on spike propagation are based on the change of VGSC’s functional status.

The fidelity of spike propagation enables the digital spikes to reach axonal terminals and trigger synaptic transmission, such that digital codes in the brain are securely propagated in neural networks. The stable velocity of spike propagation secures the temporal precision of neural codes. The amplification of incomplete spikes ensures the neuronal digital codes to reach axonal terminal [19,20]. To most neurons, these characteristics of spike propagation are beneficial to the efficient output of neuronal codes and the homeostasis of the neuronal networks. On the other hand, some neurons fire high frequency spikes, such as cerebellar Purkinje cells [9,30-32]. Their responses to *in vivo* stimulation lasted for seconds [43,44]. Persistent spikes in these neurons will intensively inhibit their downstream neurons to make them being functionally silent. The infidelity and deceleration of spike propagation as well as the failure of synaptic transmission will prevent the enhanced inhibition of their target cells. Therefore, the axons through regulating spike propagation and VGSC’s dynamics make their downstream neurons working properly, such that the brain functions are executed in the manners of precision and homeostasis.

Another physiological impact for the fidelity of spike propagation may be to make sure functional compatibility between presynaptic axons and postsynaptic neurons. The axons of cerebellar Purkinje cells sprout the branches, main axon and recurrent one. The main axon innervates neurons in deep nucleus and the recurrent axons project to adjacent Purkinje neurons [49-52]. The ability to encode digital spikes are diversified in these postsynaptic neurons [4,53-56]. The activity diversity of postsynaptic neurons require presynaptic axonal branches to be functionally differentiated. They may follow a rule that the activity levels of presynaptic axonal branches match the activity levels of their target cells, i.e., the functional compatibility between presynaptic and postsynaptic partners. In our study, the fidelity of spike propagation is higher in recurrent branches vs. main axon. The abilities of encoding spikes are advanced in Purkinje cells vs. deep nucleus cells. In addition to functional differentiations among Purkinje cell axon branches and among their target neurons, the activity strengths between presynaptic and postsynaptic partners are proportionally correlated, i.e., active axonal branches innervate active target cells, or vice versa. Computational simulation indicates that their functional compatibility makes the neurons in microcircuit being activated appropriately. Hence, each cerebellar Purkinje cell differentiates the function of its axonal branches to be compatible with the function of their target neurons in order to form a homeostatic and efficient unit [56].

The “all or none” feature of the action potentials can be understood as their identical amplitudes in spike generation and the fidelity in spike propagation. This concept is not suitable for the infidelity and deceleration of spike propagation, in which the propagation of sequential spikes on the axons is failed to reduce their number at the terminals and is decelerated to slow down neuronal encodings. For instance, spike propagation was failed on the axons of certain neurons that fire high frequency spikes [23,24,26-28]; Figures 1, 2, 3 and 4). This infidelity of spike propagation on the axons makes neuronal digital codes deteriorated, such that the messages coded in the presynaptic neurons cannot be precisely detected by the postsynaptic neurons. In order to secure neural digital codes, spike propagation is expected to be ensured. Our results indicate that the prevention of VGSC inactivation or the facilitation of VGSC reactivation improves the infidelity of spike propagation, which should be a strategy to secure the neuronal digital codes.

The membrane depolarization recorded *in vivo* was generally classified into two patterns, steady depolarization and fluctuation in synaptic integrated signals [36]. Steady depolarization inactivated VGSCs [33,35]. Hyperpolarization pulses improved VGSC’s activation [34,42]. Spikes’ generation on the fluctuated pulses is easily propagated to axonal terminals, and such less infidelity and deceleration may be useful to the neuronal encoding under the physiological condition. Spikes’ generation on steady depolarization (a more excitable state) is easily lost during their propagation on the axons, which may prevent the pathological overexcitation and save cellular energies. Thus, membrane potential and synaptic signal patterns regulate the fidelity and velocity of spike propagation in the favorable manners for neuronal encoding and seizure prevention. This functional coordination among synapses, cell bodies and axons constitutes a homeostatic process among subcellular compartments [57]. The optimal ranges for spike generation and propagation within the efficient and physiological encodings remain to be defined.

In terms of correlations between the velocity and fidelity of spike propagation, our study indicates that the propagation velocity is more easily affected by long-term depolarization and spikes (Figures 3 and 4). The decreases of spike propagation velocity fall into two steps, level one and level two (Figures 4, 7 and 9). Once the velocity of spike propagation is lowered to level two, a subsequent spike is surely failed to propagate to axonal terminals. After this failure, the velocity of spike propagation recovers to level one. The interaction of spike propagation velocity and fidelity indicate that spike propagation deceleration and infidelity share the common mechanism, i.e., VGSCs’ dynamics, in which the spike propagation velocity is more dependent of VGSC’s functions. The finding that both velocity and fidelity of spike propagation are controlled by VGSCs is advanced compared to previous studies that are focused on spike propagation fidelity or velocity [1].

What subtypes of VGSCs are involved in regulating the velocity and fidelity of spike propagation? Type I, II and VI are major type of VGSCs on the axons of the cerebellar Purkinje cells [50,58-62]. Their inactivation during sequential spikes may lead to the infidelity and deceleration of spike propagation. NaV1.1 ~ 1.2 are distributed in the proximal axons of Purkinje cells, whereas NaV1.6 is abundant at nodes of Ranvier in distal axons [62]. Therefore, NaV1.6 is most likely involved in time-dependent deceleration and infidelity of spike propagation in Purkinje cell axons, which remains to be examined once we are able to specifically manipulate NaV1.6 dynamics. It is noteworthy that the generation of action potentials needs both voltage-gated sodium channels and potassium channels. The role of potassium channels in spike propagation has not been placed in the scope of our current study. Previous studies indicated that potassium channels through affecting membrane potentials regulated the fidelity of spike propagation [24,28]. The membrane potentials influence action potentials via VGSCs [34,42]. The effect of potassium channels on spike propagation may be eventually through regulating the functional status of VGSCs, such that we focused on studying the role of VGSCs in spike propagation. The future studies should be focused on how sodium channels and potassium channels coordinately regulate the velocity and fidelity of spike propagation.

The infidelity and deceleration of spike propagation is due to VGSC inactivation. It remains to be investigated whether the processes are regulated by the intracellular signals, such as Ca^2+^/CaM-dependent protein kinases and phosphatases [57]. As the infidelity and deceleration of the axonal spike propagation occur in the late phase of depolarization-induced spikes, these spikes will more or less raise intracellular Ca^2+^. This regulation is very likely. It is noteworthy that these signal molecules regulate the function of synapses and soma [63-66]. Therefore, the encodings of synaptic analogue signals and somatic digital spikes as well as the output of axonal spike signals are commonly regulated by intracellular signaling pathways. Whether these regulations are homeostatic in nature remains be examined.

The studies in cellular imaging suggest that action potentials can reach to axonal terminals. For instance, Ca^2+^ transient was detected at both locations of somatic spike generation and transmitter release [67]. The spikes based on imaging sodium signals indicated that somatic spikes were faithfully propagated toward main axon as well as axonal collaterals in the limited frequency (<250 Hz; [50]. These results were obtained from the studies in a single spike propagation or proximal axons. This suggestion may not be suitable for sequential spikes and their propagation to axonal terminals. In addition, one could argue that the axons might be injured during cutting cerebellar slices. As the infidelity of spike propagation can be almost reversed by afterhyperpolarization to reactivate VGSCs (Figure 6), this argument may not be an issue.

## Methods and materials

### Brain slices and neurons

All experiments were approved by the Institutional Animal Care Unit Committee in Administration Office of Laboratory Animals Beijing China (B10831). Cerebellar sagittal slices (400 μm) were prepared from Wistar rats in postnatal day 14 ~ 15 that were anesthetized by injecting chloral hydrate (300 mg/kg) and decapitated by a guillotine. The slices were cut by a Vibratome in the modified and oxygenized (95% O_2_ and 5% CO_2_) artificial cerebrospinal fluid (mM: 124 NaCl, 3 KCl, 1.2 NaH_2_PO_4_, 26 NaHCO_3_, 0.5 CaCl_2_, 5 MgSO_4_, 10 dextrose and 5 HEPES; pH 7.4) at 4°C, and were held in the normal oxygenated ACSF (mM: 126 NaCl, 2.5 KCl, 1.25 NaH_2_PO_4_, 26 NaHCO_3_, 2 CaCl_2_, 2 MgSO_4_ and 25 dextrose; pH 7.4) at 35°C for 1–2 hours. A slice was transferred to a submersion chamber (Warner RC-26G) and perfused by normal ACSF for electrophysiological experiments [57,68-70].

Cerebellar Purkinje cells were identified based on their morphology and functions. Purkinje cells (somata above 40 μm) in the slices for whole-cell recording were located at the border between molecular layer and granule cells, and infused by fluorescence Alex-488 (5 μM in pipettes) under a DIC/fluorescent microscope (Nikon, FN-E600). The excited fluorophore showed the typical dendrites and axonal branches of Purkinje cells, through which we traced their main axons for recording spikes by loose-patch. Purkinje cells also were labeled by neurobiotin (Figure 1A). Purkinje cells showed fast spiking and no adaptation in amplitude and frequency [18,42,71-75].

### Electrophysiological studies

Sequential spikes in Purkinje cells propagate on their main axons. The experiments were conducted by whole-cell recordings on their somata to induce spikes and by loose-patch recordings on the remote ends of their main axons (Figure 1A) to record the propagated spikes. The electrical signals were recorded by a MultiClapm-700B amplifier (Axon Instrument Inc, CA USA) and inputted into a pClamp-10 with 50 kHz sampling rate. The transient capacitance was compensated and output bandwidth was 3 kHz. The pipette solution for recording spikes in whole-cell model included (mM) 150 K-gluconate, 5 NaCl, 0.4 EGTA, 4 Mg-ATP, 0.5 Tris- GTP, 4 Na-phosphocreatine and 10 HEPES (pH 7.4 adjusted by 2 M KOH). The solution for axonal loose-patch recording was ACSF. The osmolarity of pipette solution made freshly was 295–305 mOsmol, and pipette resistance was 8 ~ 10 MΩ.

In the study of spike propagation on the main axons of Purkinje cells, we injected depolarization pulses in various durations and intervals into the somata to induce the spikes at 100 ~ 200 Hz, and recorded spike propagation at the remote ends of their main axons. In addition to fluorescent tracing, the spikes at soma and axonal bleb with phase-locking indicated the signals from a Purkinje cell. The fidelity of spike propagation on the axonal branches of Purkinje cells was assessed by a ratio of spikes recorded at axonal terminals to spikes induced on somata. The velocity of spike propagation on the axons of Purkinje cells was calculated by the formula that the lengths from somata to axonal blebs were divided by the peak-time intervals between axonal spikes and somatic ones, in which the somatic spikes were converted into dV/dt [17,76]. As spike propagation was time-dependent, the fidelity and velocity of spike propagation at every 50 ms were averaged from ten spikes. This calculation method was also used to quantify spike-rising slope (maximal dV/dt). As the lengths of axons may be variable in our experiments, the presentation of spike propagation velocity was normalized.

The influence of VGSC’s functional status on spike propagation was studied. The inactivation of VGSCs was prevented by using anemone toxin (ATX), a blocker of VGSC inactivation [45,46], or hyperpolarization pulses [34,42]. 5 μM ATX was puffed onto the middle segment of main axons by ATX-containing pipette, while using whole-cell recording on soma and loose-patch recording on axonal bleb. The inactivation of VGSCs was made by using a steady depolarization [34,42].

The data were analyzed if the recorded neurons had resting membrane potentials negatively more than −60 mV and action potentials above 70 mV. The criteria for the acceptation of each experiment also included less than 5% changes in resting membrane potential, spike magnitude, input and seal resistance. The values of the spike propagation velocity, fidelity and maximal dV/dt are presented as mean ± SE. The comparisons between groups are done by t-test.

### Neurobiotin staining for cerebellar cells

Pipette solutions for whole-cell recordings included 0.2% neurobiotin, which were back-filled into the recording pipettes whose tips contained the standard solution. After electrophysiological study, the slices were rapidly placed into 4% paraformaldehyde in 0.1 M phosphate buffer solution (PBS) for fixation at 4°C about 48 hours. The slices were incubated in avidin and horseradish peroxidase (Vectastain ABC) for 3 hours, and then 1% DAB–CoCl2 (Sigma) 1 min for staining neurobiotin-filled cells. This reaction was stopped by PBS [77]. Neurobiotin-stained cells were photographed under a scanning confocal microscope (Olympus FV-1000, Japan).

## Competing interests

All authors declare that they have no competing interests.

## Authors’ contributions

ZY at al. work on the experiments and data analyses. JHW contributes to the project design and paper writing. All authors read and approved the final manuscript.

## References

[B1] Debanne D Campanac E Bialowas A Carlier E Alcaraz G Axon physiology Physiol Rev 2011 91 555 602 10.1152/physrev.00048.2009 21527732

[B2] Kandel ER Siegelbaum SA Schwartz JH Kandel ER, Schwartz JH, Jessell TM Elementary interactions between neurons: synaptic transmission Principles of neural science 2000 3 New York: McGraw-Hill 175 308

[B3] Shepherd GM Shepherd GM Synaptic transmission Neurobiology 1998 4 New York: Oxford University Press

[B4] Wang JH Wei J Chen X Yu J Chen N Shi J The gain and fidelity of transmission patterns at cortical excitatory unitary synapses improve spike encoding J Cell Sci 2008 121 2951 2960 10.1242/jcs.025684 18697836

[B5] Yu J Qian H Chen N Wang JH Quantal glutamate release is essential for reliable neuronal encodings in cerebral networks PLoS ONE 2011 6 e25219 10.1371/journal.pone.0025219 21949885 PMC3176814

[B6] Clark BA Monsivais P Branco T London M Hausser M The site of action potential initiation in cerebellar Purkinje neurons Nat Neurosci 2005 8 137 139 10.1038/nn1390 15665877

[B7] Colbert CM Pan E Ion channel properties underlying axonal action potential initiation in pyramidal neurons Nat Neurosci 2002 5 533 538 10.1038/nn0602-857 11992119

[B8] Coombs JS Curtis DR Eccles JC The interpretation of spike potentials of motoneurones J Physiol 1957 139 198 231 13492209 10.1113/jphysiol.1957.sp005887 PMC1358725

[B9] Davie JT Clark BA Hausser M The origin of the complex spike in cerebellar Purkinje cells J Neurosci 2008 28 7599 7609 10.1523/JNEUROSCI.0559-08.2008 18650337 PMC2730632

[B10] Fuortes MGF Frank K Becker MC Steps in the production of motor neuron spikes J Gen Physiol 1957 40 735 752 10.1085/jgp.40.5.735 13428986 PMC2147645

[B11] Hausser M Stuart G Racca C Sakmann B Axonal initiation and active dendritic propagation of action potentials in substantia nigra neurons Neuron 1995 15 637 647 10.1016/0896-6273(95)90152-3 7546743

[B12] Hu W Tian C Li T Yang P Hou H Shu YS Distinct contribution of Nav1.6 and Nav1.2 in action potential initiation and backpropagation Nat Neurosci 2009 12 996 1002 10.1038/nn.2359 19633666

[B13] Kang Y Saito M Sato H Toyoda H Maeda Y Hirai T Bae YC Involvement of persistent Na + current in spike initiation in primary sensory neurons of the rat mesencephalic trigeminal nucleus J Neurophysiol 2007 97 2385 2393 10.1152/jn.01191.2006 17229822

[B14] Kole MHP Ilschner SU Kampa BM Williams SR Ruben PC Stuart GJ Action potential generation requires a high sodium channel density in the axon initial segment Nat Neurosci 2008 11 178 186 10.1038/nn2040 18204443

[B15] Kuba H Ishii TM Ohmori H Axonal site of spike initiation enhances auditory coincidence detection Nature 2006 444 1069 1072 10.1038/nature05347 17136099

[B16] Maarten H Kole P Stuart GJ Is action potential threshold lowest in the axon? Nat Neurosci 2008 11 1253 1255 10.1038/nn.2203 18836442

[B17] Meeks JP Mennerick S Action potential initiation and propagation in CA3 pyramidal axons J Neurophysiol 2007 97 3460 3472 10.1152/jn.01288.2006 17314237

[B18] Palmer LM Clark BA Grundemann J Roth A Stuart GJ Hausser M Initiation of simple and complex spikes in cerebellar Purkinje cells J Physiol 2010 588 1709 1717 10.1113/jphysiol.2010.188300 20351049 PMC2887989

[B19] Chen N Yu J Qian H Ge R Wang JH Axons amplify somatic incomplete spikes into uniform amplitudes in mouse cortical pyramidal neurons PLoS ONE 2010 5 7 e11868 10.1371/journal.pone.0011868 20686619 PMC2912328

[B20] Engel D Jonas P Presynaptic action potential amplification by voltage-gated Na + channels in hippocampal mossy fiber boutons Neuron 2005 45 405 417 10.1016/j.neuron.2004.12.048 15694327

[B21] Hodgkin AL Huxley AF Propagation of electrical signals along giant nerve fibers Proc R Soc Lond B Biol Sci 1952 140 177 183 10.1098/rspb.1952.0054 13003922

[B22] Huxley AF Stampfli R Evidence for saltatory conduction in peripheral myelinated nerve fibres J Physiol 1949 108 315 339 PMC1392492 18144923

[B23] Khaliq ZM Raman IM Axonal propagation of simple and complex spikes in cerebellar Purkinje neurons J Neurosci 2005 25 454 463 10.1523/JNEUROSCI.3045-04.2005 15647489 PMC6725469

[B24] Monsivais P Clark BA Roth A Hausser M Determinants of action potential propagation in cerebellar Purkinje cell axons J Neurosci 2005 25 464 472 10.1523/JNEUROSCI.3871-04.2005 15647490 PMC6725482

[B25] Bucher D Goaillard JM Beyond faithful conduction: short-term dynamics, neuromodulation, and long-term regulation of spike propagation in the axon Prog Neurobiol 2011 94 307 346 10.1016/j.pneurobio.2011.06.001 21708220 PMC3156869

[B26] Debanne D Guerineau NC Gahwiler BH Thompson SM Action-potential propagation gated by an axonal I (A)-like K + conductance in hippocampus Nature 1997 389 286 289 10.1038/38502 9305843

[B27] Khaliq ZM Raman IM Relative contributions of axonal and somatic Na channels to action potential initiation in cerebellar Purkinje neurons J Neurosci 2006 26 1935 1944 10.1523/JNEUROSCI.4664-05.2006 16481425 PMC6674931

[B28] Meeks JP Mennerick S Selective effects of potassium elevations on glutamate signaling and action potential conduction in hippocampus J Neurosci 2004 24 197 206 10.1523/JNEUROSCI.4845-03.2004 14715952 PMC6729587

[B29] Debanne D Information processing in the axon Nat Rev Neurosci 2004 5 304 316 10.1038/nrn1397 15034555

[B30] Eccles JC Sasaki K Strata P Interpretation of the potential fields generated in the cerebellar cortex by a mossy fibre volley Exp Brain Res 1967 3 58 80 6031000 10.1007/BF00234470

[B31] Harvey RJ Porter R Rawson JA The natural discharges of Purkinje cells in paravermal regions of lobules V and VI of the monkey’s cerebellum J Physiol 1977 271 515 536 411917 10.1113/jphysiol.1977.sp012012 PMC1353584

[B32] Llinas R Sugimori M Electrophysiological properties of in vitro Purkinje cell somata in mammalian cerebellar slices J Physiol 1980 305 171 195 7441552 10.1113/jphysiol.1980.sp013357 PMC1282966

[B33] Aldrich RW Corey DP Stevens CF A reinterpretation of mammalian sodium channel gating based on single channel recording Nature 1983 306 436 441 10.1038/306436a0 6316158

[B34] Chen N Chen X Yu J Wang J-H After-hyperpolarization improves spike programming through lowering threshold potentials and refractory periods mediated by voltage-gated sodium channels Biochem Biophys Res Commun 2006 346 938 945 10.1016/j.bbrc.2006.06.003 16777065

[B35] Goldman L Stationarity of sodium channel gating kinetics in excised patches from neuroblastoma N1E 115 Biophysics Journal 1995 69 2364 2368 10.1016/S0006-3495(95)80105-0 PMC1236473 8599642

[B36] Ge R Qian H Wang JH Physiological synaptic signals initiate sequential spikes at soma of cortical pyramidal neurons Mol Brain 2011 4 19 10.1186/1756-6606-4-19 21549002 PMC3113741

[B37] Deqenetais E Thierry AM Glowinski J Gioanni Y Electrophysiological properties of pyramidal neurons in the rat prefrontal cortex: an in vivo intracellular recording study Cereb Cortex 2002 12 1 16 10.1093/cercor/12.1.1 11734528

[B38] Haider B Duque A Hasenstaub A McCormick DA Neocortical network activity in vivo is generated through a dynamic balance of excitation and inhibition J Neurosci 2006 26 4535 4545 10.1523/JNEUROSCI.5297-05.2006 16641233 PMC6674060

[B39] Henze DA Buzsaki G Action potential threshold of hippocampal pyramidal cells in vivo is increased by recent spiking activity Neuroscience 2001 105 121 130 10.1016/S0306-4522(01)00167-1 11483306

[B40] Zhang Z Yu YQ Liu CH Chan YS He J Reprint of “frequency tuning and firing pattern properties of auditory thalamic neurons: an in vivo intracellular recording from the guinea pig” Neuroscience 2008 154 273 282 10.1016/S0306-4522(08)00741-0 18555163

[B41] Chen N Chen SL Wu YL Wang JH The refractory periods and threshold potentials of sequential spikes measured by whole-cell recordings Biochem Biophys Res Commun 2006 340 151 157 10.1016/j.bbrc.2005.11.170 16343428

[B42] Chen N Zhu Y Gao X Guan S Wang J-H Sodium channel-mediated intrinsic mechanisms underlying the differences of spike programming among GABAergic neurons Biochem Biophys Res Commun 2006 346 281 287 10.1016/j.bbrc.2006.05.120 16756951

[B43] Jaeger D Bower JM Prolonged responses in rat cerebellar Purkinje cells following activation of the granule cell layer: an intracellular in vitro and in vivo investigation Exp Brain Res 1994 100 200 214 7813659 10.1007/BF00227191

[B44] Loewenstein Y Mahon S Chadderton P Kitamura K Sompolinsky H Yarom Y Hausser M Bistability of cerebellar Purkinje cells modulated by sensory stimulation Nat Neurosci 2005 8 202 211 10.1038/nn1393 15665875

[B45] Mantegazza M Franceschetti S Avanzini G Anemone toxin (ATX II)-induced increase in persistent sodium current: effects on the firing properties of rat neocortical pyramidal neurones J Physiol 1998 507 Pt 1 105 116 9490824 10.1111/j.1469-7793.1998.105bu.x PMC2230778

[B46] Rathmayer W Anemone toxin discriminates between ionic channels for receptor potential and for action potential production in a sensory neuron Neurosci Lett 1979 13 313 318 10.1016/0304-3940(79)91512-X 43492

[B47] Berecki G Wilders R de Jonge B van Ginneken AC Verkerk AO Re-evaluation of the action potential upstroke velocity as a measure of the Na + current in cardiac myocytes at physiological conditions PLoS ONE 2010 5 e15772 10.1371/journal.pone.0015772 21217835 PMC3013114

[B48] Remme CA Verkerk AO Nuyens D van Ginneken AC van Brunschot S Belterman CN Wilders R van Roon MA Tan HL Wilde AA Overlap syndrome of cardiac sodium channel disease in mice carrying the equivalent mutation of human SCN5A-1795insD Circulation 2006 114 2584 2594 10.1161/CIRCULATIONAHA.106.653949 17145985

[B49] D’Angelo E Mazzarello P Prestori F Mapelli J Solinas S Lombardo P Cesana E Gandolfi D Congi L The cerebellar network: from structure to function and dynamics Brain Res Rev 2011 66 5 15 10.1016/j.brainresrev.2010.10.002 20950649

[B50] Foust A Popovic M Zecevic D McCormick DA Action potentials initiate in the axon initial segment and propagate through axon collaterals reliably in cerebellar Purkinje neurons J Neurosci 2010 30 6891 6902 10.1523/JNEUROSCI.0552-10.2010 20484631 PMC2990270

[B51] Sugihara I Fujita H Na J Quy PN Li BY Ikeda D Projection of reconstructed single Purkinje cell axons in relation to the cortical and nuclear aldolase C compartments of the rat cerebellum J Comp Neurol 2009 512 282 304 10.1002/cne.21889 19003905

[B52] Wang DJ Yang D Su LD Xie YJ Zhou L Sun CL Wang Y Wang XX Shen Y Cytosolic phospholipase A2 alpha/arachidonic acid signaling mediates depolarization-induced suppression of excitation in the cerebellum PLoS ONE 2012 7 e41499 10.1371/journal.pone.0041499 22927908 PMC3425552

[B53] Markram H Toledo-Rodriguez M Wang Y Gupta A Silbergerg G Wu C Interneurons of the neocortical inhibitory system Nature Review of Neuroscience 2004 5 793 807 10.1038/nrn1519 15378039

[B54] Plazas PV Nicol X Spitzer NC Activity-dependent competition regulates motor neuron axon pathfinding via PlexinA3 Proc Natl Acad Sci U S A 2013 110 1524 1529 10.1073/pnas.1213048110 23302694 PMC3557035

[B55] Uusisaari M Knopfel T Functional classification of neurons in the mouse lateral cerebellar nuclei Cerebellum 2011 10 637 646 10.1007/s12311-010-0240-3 21116763 PMC3215887

[B56] Wang JH Yang Z Qian H Chen N Functional compatibility between Purkinje cell axon branches and their target neurons in the cerebellum Biophys J 2013 104 330a 10.18632/oncotarget.19770 PMC5641142 29069799

[B57] Chen N Chen X Wang J-H Homeostasis established by coordination of subcellular compartment plasticity improves spike encoding J Cell Sci 2008 121 2961 2971 10.1242/jcs.022368 18697837

[B58] Caldwell JH Schaller KL Lasher RS Peles E Levinson SR Sodium channel Na (v)1.6 is localized at nodes of ranvier, dendrites, and synapses Proc Natl Acad Sci U S A 2000 97 5616 5620 10.1073/pnas.090034797 10779552 PMC25877

[B59] Carter BC Bean BP Incomplete inactivation and rapid recovery of voltage-dependent sodium channels during high-frequency firing in cerebellar Purkinje neurons J Neurophysiol 2011 105 860 871 10.1152/jn.01056.2010 21160003 PMC3059179

[B60] Garrido JJ Fernandes F Moussif A Fache MP Giraud P Dargent B Dynamic compartmentalization of the voltage-gated sodium channels in axons Biol Cell 2003 95 437 445 10.1016/S0248-4900(03)00091-1 14597261

[B61] Schaller KL Caldwell JH Expression and distribution of voltage-gated sodium channels in the cerebellum Cerebellum 2003 2 2 9 10.1080/14734220309424 12882229

[B62] Waxman SG Cummins TR Black JA Dib-Hajj S Diverse functions and dynamic expression of neuronal sodium channels Novartis Found Symp 2002 241 34 60 11771649

[B63] Wang JH Kelly PT Postsynaptic injection of Ca2+/CaM induces synaptic potentiation requiring CaM-KII and PKC activity Neuron 1995 15 443 452 10.1016/0896-6273(95)90048-9 7646896

[B64] Wang J-H Kelly PT Balance between postsynaptic Ca2 + −dependent protein kinase and phosphatase activities controlling synaptic strength Learn Mem 1996 3 170 181 10.1101/lm.3.2-3.170 10456087

[B65] Wang J-H Kelly PT Postsynaptic calcineurin activity down-regulates synaptic transmission by weakening intracellular Ca2+ signaling mechanisms in hippocampal CA1 neurons J Neurosci 1997 17 4600 4611 9169521 10.1523/JNEUROSCI.17-12-04600.1997 PMC6573351

[B66] Zhang M Hung F Zhu Y Xie Z Wang J Calcium signal-dependent plasticity of neuronal excitability developed postnatally J Neurobiol 2004 61 277 287 10.1002/neu.20045 15382030

[B67] Mackenzie PJ Umemiya M Murphy TH Ca2+ imaging of CNS axons in culture indicates reliable coupling between single action potentials and distal functional release sites Neuron 1996 16 783 795 10.1016/S0896-6273(00)80098-7 8607996

[B68] Ge R Chen N Wang JH Real-time neuronal homeostasis by coordinating VGSC intrinsic properties Biochem Biophys Res Commun 2009 387 585 589 10.1016/j.bbrc.2009.07.066 19616515

[B69] Wang J-H Short-term cerebral ischemia causes the dysfunction of interneurons and more excitation of pyramidal neurons Brain Res Bull 2003 60 53 58 10.1016/S0361-9230(03)00026-1 12725892

[B70] Yu J Qian H Wang JH Upregulation of transmitter release probability improves a conversion of synaptic analogue signals into neuronal digital spikes Mol Brain 2012 5 26 10.1186/1756-6606-5-26 22852823 PMC3497613

[B71] Hockberger PE Tseng H-Y Connor JA Development of rat cerebellar purkinje cells: electrophysiological properties following acute isolation and in long-term culture J Neurosci 1989 9 2258 2271 2746328 10.1523/JNEUROSCI.09-07-02258.1989 PMC6569754

[B72] McKay BE Turner RW Physiological and morphological development of the rat cerebellar Purkinje cell J Physiol Lond 2005 567 Pt3 829 850 16002452 10.1113/jphysiol.2005.089383 PMC1474219

[B73] Qi Y Huang L Ni H Zhou X Zhang J Zhu Y Ge M Guan S Wang JH Intracellular Ca2+ regulates spike encoding at cortical GABAergic neurons and cerebellar Purkinje cells differently Biochem Biophys Res Commun 2009 381 129 133 10.1016/j.bbrc.2009.02.058 19351606

[B74] Stuart G Hausser M Initiation and spread of sodium action potentials in cerebellar Purkinje cells Neuron 1994 13 703 712 10.1016/0896-6273(94)90037-X 7917300

[B75] Wang JH Zhang M Differential modulation of glutamatergic and cholinergic synapses by calcineurin in hippocampal CA1 fast-spiking interneurons Brain Res 1004 125 135 15033427 10.1016/j.brainres.2004.01.025

[B76] Kress GJ Dowling MJ Meeks JP Mennerick S High threshold, proximal initiation, and slow conduction velocity of action potentials in dentate granule neuron mossy fibers J Neurophysiol 2008 100 281 291 10.1152/jn.90295.2008 18480368 PMC2493481

[B77] Wang J-H Kelly PT Ca2+/CaM signalling pathway up-regulates glutamatergic synaptic function in non-pyramidal fast-spiking neurons of hippocampal CA1 J Physiol Lond 2001 533 407 422 10.1111/j.1469-7793.2001.0407a.x 11389201 PMC2278630

